# CFTR-NHERF2-LPA_2_ Complex in the Airway and Gut Epithelia

**DOI:** 10.3390/ijms18091896

**Published:** 2017-09-04

**Authors:** Weiqiang Zhang, Zhihong Zhang, Yanhui Zhang, Anjaparavanda P. Naren

**Affiliations:** 1 Department of Pediatrics & Department of Physiology, College of Medicine, University of Tennessee Health Science Center, Memphis, TN 38103, USA; zzhang19@uthsc.edu; 2 Department of Bioscience research, College of Dentistry, University of Tennessee Health Science Center, Memphis, TN 38163, USA; yzhang36@uthsc.edu; 3 Department of Pediatrics, Cincinnati Children’s Hospital Medical Center, Cincinnati, OH 45229, USA

**Keywords:** ABC transporters, CFTR, NHERF2, LPA_2_, macromolecular protein complex, cystic fibrosis, secretory diarrhea

## Abstract

The cystic fibrosis transmembrane conductance regulator (CFTR) is a cAMP- and cGMP-regulated chloride (Cl^−^) and bicarbonate (HCO_3_^−^) channel localized primarily at the apical plasma membrane of epithelial cells lining the airway, gut and exocrine glands, where it is responsible for transepithelial salt and water transport. Several human diseases are associated with altered CFTR channel function. Cystic fibrosis (CF) is caused by the absence or dysfunction of CFTR channel activity, resulting from mutations in the gene. Secretory diarrhea is caused by the hyperactivation of CFTR channel activity in the gastrointestinal tract. CFTR is a validated target for drug development to treat CF, and extensive research has been conducted to develop CFTR inhibitors for therapeutic interventions of secretory diarrhea. The intracellular processing, trafficking, apical membrane localization, and channel function of CFTR are regulated by dynamic protein–protein interactions in a complex network. In this paper, we review the current knowledge of a macromolecular complex of CFTR, Na^+^/H^+^ exchanger regulatory factor 2 (NHERF2), and lysophosphatidic acids (LPA) receptor 2 (LPA_2_) at the apical plasma membrane of airway and gut epithelial cells, and discuss its relevance in human physiology and diseases. We also explore the possibilities of targeting this complex to fine tune CFTR channel activity, with a hope to open up new avenues to develop novel therapies for CF and secretory diarrhea.

## 1. Introduction

Protein–protein interactions regulate virtually all cellular processes by promoting the proper cellular localization of regulatory partners and by facilitating the signaling through pathways to achieve exquisite spatiotemporal control. The formation of multiple protein complexes at discrete subcellular microdomains increases the specificity and efficiency of cell signaling [1,2,3].

**Figure 1:**
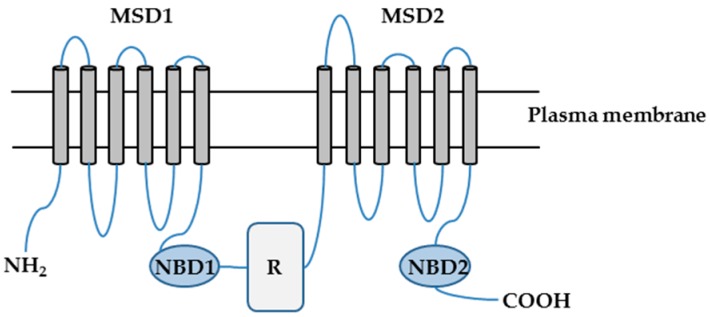
The putative domain structure of the cystic fibrosis transmembrane conductance regulator (CFTR) protein. CFTR is composed of two membrane-spanning domains (MSD1 and MSD2), two nucleotide binding domains (NBD1 and NBD2), and a regulatory domain (R). NH_2_: amino terminal tail; COOH: carboxyl terminal tail.

CFTR is a cAMP- and cGMP-regulated chloride (Cl^−^) and bicarbonate (HCO_3_^−^) channel localized primarily at the apical surfaces of epithelial cells lining the airways, gut, and exocrine glands, where it is responsible for the transepithelial salt and water transport [4,5,6]. CFTR is a member of the ATP-binding cassette (ABC) transporter superfamily and consists of 1480 amino acids. CFTR is composed of two repeated motifs; each consisting of a six-helix membrane-spanning domain (MSD) and a cytosolic nucleotide binding domain (NBD). These two motifs are linked by a cytoplasmic regulatory (R) domain, which contains multiple consensus phosphorylation sites (Figure 1) [4,7,8].

The R domain is a unique feature of CFTR within the ABC superfamily. Both the amino and carboxyl termini of CFTR mediate its interactions with a wide variety of binding partners [9]. CFTR channel is activated by phosphorylation of its R domain and binding and hydrolysis of ATP at NBDs. CFTR channel activity is determined by the quantity of channels at the plasma membrane, the open probability of these channels, and their single channel conductance. Mutations in the *CFTR* gene alter one or more of these parameters, causing the impairment or loss of the channel activity. More than 2000 mutations have been identified in the *CFTR* gene [10], which are traditionally grouped into six classes based on the nature of the defect(s) [9,10]. Class I mutations have defects in biosynthesis, resulting in low levels of truncated and/or dysfunctional CFTR proteins. Class II mutations have defects in folding or maturation, causing no to very little CFTR protein to reach the cell surface. Class III mutations encode CFTR proteins that have defects in channel gating, and Class IV mutations encode proteins that have reduced capacity to transport Cl^−^. Class V mutations have reduced mRNA stability. Class VI mutations encode CFTR proteins with decreased stability and increased turnover at the cell surface [11,12]. Because some mutations have multiple defects, an expanded classification method was also proposed [13]. One such mutation is Phe508del (deletion of a phenylalanine residue at position 508 on CFTR protein), which is the most prevalent CFTR mutation with approximately 90% of CF patients carrying it on at least one allele. Phe508del is a class II mutation. However, upon reaching the cell surface following rescue procedures, it displays characteristics of class III and VI mutations [13].

The intracellular processing, trafficking, apical plasma membrane localization and channel function of CFTR are regulated by dynamic protein–protein interactions in a complex network (CFTR interactome). A wide variety of CFTR-interacting partners have been identified, including receptors, scaffolding proteins, channels, transporters, etc. [9,14]. Several CFTR-containing macromolecular complexes at the apical plasma membrane of epithelial cells have been characterized; examples include (i) the complex of β_2_-adrenergic receptor (β_2_-AR), Na^+^/H^+^ exchanger regulatory factor 1, and CFTR at the apical surfaces of airway epithelial cells, which couples β_2_-AR signaling to CFTR channel function [15], (ii) the complex of multidrug resistance protein 4 (MRP4), PDZ-containing kidney protein 1, and CFTR at the apical surfaces of intestinal epithelial cells, which couples the cAMP transporter activity of MRP4 to CFTR channel function [3], and (iii) the complex of LPA_2_, NHERF2, and CFTR at the apical surfaces of airway and intestinal epithelial cells, which couples the LPA_2_-mediated signaling to CFTR channel function [16]. In this article, we review the current knowledge of CFTR-NHERF2-LPA_2_ complex at the apical plasma membrane of airway and gut epithelial cells and its relevance in human physiology and diseases. We also explore the possibilities, and provide our perspectives, on how to target this complex to fine tune CFTR channel activity, with a hope to open up new avenues to develop novel therapeutics for CFTR-associated diseases.

## 2. CFTR-NHERF2-LPA_2_ Complex in Airway and Gut Epithelial Cells

### 2.1. Characterization of CFTR-NHERF2-LPA_2_ Complex

NHERF2 is a postsynaptic density-95, discs large, zona occludens-1 (PDZ) domain-containing protein and primarily localizes at the apical plasma membrane of epithelial cells. NHERF2 has 337 amino acids and contains two PDZ domains and an ezrin/radixin/moesin (ERM) domain at the C-terminus. The ERM domain mediates the interaction of NHERF2 with merlin/ERM proteins and links NHERF2 to the actin cytoskeleton [17]. NHERF2 has been shown to cluster signaling molecules into macromolecular complexes [9,16,17,18,19].

LPA are growth-factor-like phospholipids present in every tissue and most biological fluids at nanomolar to micromolar concentrations. LPA mediate diverse cellular responses, such as proliferation, migration, survival, angiogenesis, inflammation and much more [20]. At least six G-protein-coupled LPA receptors have been identified, which couple to G_s_, G_i_, G_q_ and/or G_12/13_ to activate various signaling pathways [20,21]. The LPA receptor LPA_2_ contains 351 amino acids and belongs to the endothelial differentiation gene family. LPA_2_ is structurally unique at the C-terminus, in which it contains a dileucine motif and several putative palmitoylated cysteine residues in the proximal region that are responsible for binding to several zinc-finger proteins [22,23]. The last four amino acids of LPA_2_, Asp-Ser-Thr-Leu (DSTL), form a class I PDZ domain-binding motif and mediate the interaction of LPA_2_ with several PDZ proteins, including NHERF2 [23]. Through the interaction with LPA_2_, NHERF2 regulates the LPA-mediated phospholipase C-β3 (PLC-β3) signaling pathway and the activation of extracellular signal-regulated kinases and Akt [24,25]. LPA has been reported to induce the formation of a ternary complex containing LPA_2_, thyroid-hormone-receptor-interacting protein 6, and NHERF2 at microdomains on the plasma membrane, which regulates the anti-apoptotic signaling of LPA_2_ [26].

**Figure 2:**
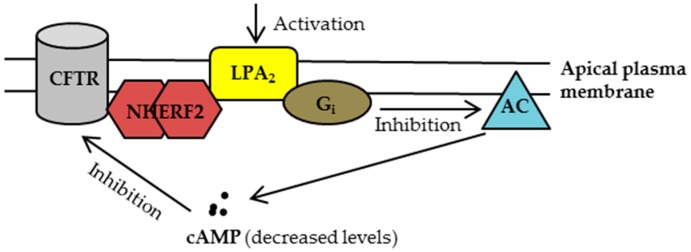
Once residing at the plasma membrane, the cystic fibrosis transmembrane conductance regulator (CFTR) forms a complex with Na^+^/H^+^ exchanger regulatory factor 2 (NHERF2) and lysophosphatidic acid receptor 2 (LPA_2_), which couples the LPA_2_-mediated signaling with CFTR channel activity in a compartmentalized manner. Upon activation of LPA_2_, adenylyl cyclase (AC) is inhibited through G_i_ pathway, leading to a decreased cAMP level in proximity to CFTR and consequently inhibiting CFTR channel function. For clarity, only the major signaling molecules involved in this macromolecular complex are depicted here.

Li et al. [16] demonstrated for the first time that (i) wild-type (WT)-CFTR forms a macromolecular complex with NHERF2 and LPA_2_ at the apical plasma membrane of intestinal epithelial cells (HT29-CL19A) and airway epithelial cells (Calu-3); (ii) LPA inhibits the CFTR Cl^−^ channel function (using a CFTR-mediated iodide efflux assay) through an LPA_2_-mediated G_i_ pathway; (iii) LPA inhibits the CFTR-dependent short-circuit currents (I_sc_) in polarized epithelial cells in a compartmentalized manner; (iv) LPA inhibits the CFTR-dependent I_sc_ in mouse intestinal epithelia tissues; (v) Administration of LPA substantially reduced the cholera toxin (CTX)-induced and CFTR-mediated intestinal fluid secretion in mice; and (vi) disruption of this complex using an LPA_2_-specific peptide, which contains the last 11 amino acids at the C-terminus of LPA_2_ and serves as a disruptor of LPA_2_-NHERF2 interaction in cells, reversed the LPA_2_-mediated inhibition of CFTR channel function in cells [16]. This study not only discovered the molecular mechanism underlying the coupling of LPA_2_-mediated inhibitory signaling to CFTR Cl^−^ channel function at the apical plasma membrane of airway and intestinal epithelial cells (Figure 2), but demonstrated that LPA substantially reduced the CTX-induced intestinal fluid secretion in vivo, which had clinical implications for treating human diseases associated with the hyperactivation of CFTR channel function (e.g., secretory diarrhea).

In a study to investigate the roles of NHERF1/2/3 in regulating the CFTR-dependent duodenal HCO_3_^−^ secretion in mice, Singh et al. found that the forskolin (FSK)-stimulated HCO_3_^−^ secretion was significantly increased in *Nherf*2^−^/^−^ mice and that NHERF2 is required for the LPA_2_-mediated inhibition of HCO_3_^−^ secretion [27]. The data supported the findings of Li and colleagues’ [16] and together suggested that by attenuating this LPA_2_-mediated inhibitory signaling, CFTR channel function could be potentially augmented.

Phe508del-CFTR protein has multiple defects, including a folding defect which results in the protein to get trapped in the endoplasmic reticulum and targeted for degradation, with little to no protein trafficked to the cell surface. Although the mutant protein can be partially rescued by exposure to low temperature and/or using CFTR correctors, it is unstable at the cell surface and exhibits impaired channel activity [11,12,28]. Recently, we found that when rescued to the plasma membrane, Phe508del-CFTR also complexes with NHERF2 and LPA_2_ in CF bronchial epithelial cells (CFBEo^−^-Phe508del-CFTR cells) and in intestinal enterospheres developed from *Phe508del^−/−^* mice [29]. By formation of such a complex, the LPA_2_-mediated signaling could exert its inhibitory effect on the rescued Phe508del-CFTR at the cell surface (Figure 2). 

### 2.2. The Involvment of CFTR in Two Major Human Diseases: Cystic Fibrosis (CF) and Secretory Diarrhea

Several human diseases are associated with altered channel function of CFTR, including CF and secretory diarrhea [9,30]. 

CF is a life-shortening autosomal recessive inherited disease caused by the absence or dysfunction of CFTR channel activity, resulting from mutations in the *CFTR* gene [31,32]. There are approximately 70,000 CF patients worldwide [33]. Clinically, CF affects multiple organs, including the lungs (chronic lung disease causes most of the CF-associated morbidity and mortality), upper airway (e.g., sinusitis), pancreas (e.g., pancreatic insufficiency, CF-related diabetes mellitus), sweat glands (elevated sweat chloride level), intestines (e.g., meconium ileus, constipation, distal intestinal obstruction syndrome), liver (e.g., cholestasis, cirrhosis), and vas deferens (male infertility) [34]. In the CF lungs, the loss of CFTR function causes a cascade of pathological events: the depletion of airway surface liquid (ASL), mucus plugging the airways, failure of mucociliary clearance, chronic bacterial infections, and excessive and ineffective inflammation (which fails to eradicate pulmonary pathogens). This may cause bronchiectasis and progressive airway destruction, eventually leading to the loss of pulmonary function [28,35]. Other genetic and environmental factors (e.g., modifier genes, socioeconomic status) also strongly influence the severity of the disease [35]. LPA levels have been found elevated in the bronchoalveolar lavage (BAL) fluid of subjects with CF [36], suggesting that LPA and its receptors may play a role in the pathogenesis of CF. 

Secretory diarrhea involves the hyperactivation of CFTR channel in the gastrointestinal tract [37]. When the gut lumen is exposed to certain types of stimuli (e.g., toxins secreted by the colonizing pathogenic microorganisms *Escherichia coli*, *Vibrio cholera* [38]), the intracellular second messengers cAMP and/or cGMP are excessively produced, causing the hyperactivation of CFTR channel. This hyperactivation increases the electrical and osmotic driving forces for the parallel flows of Na^+^ and water and inhibits the fluid absorption processes mediated by Na^+^/H^+^ exchangers (e.g., NHE3) and epithelial sodium channel. The net result is the excessive fluid secretion into the intestine lumen, which overwhelms the reabsorbing capacity of the colon and leads to fluid loss and dehydration [9,30]. Because CFTR is the primary chloride channel at the apical membrane of intestinal epithelial cells and plays a critical role in intestinal fluid secretion and homeostasis, extensive research has been conducted to develop CFTR inhibitors (channel blockers) as potential anti-diarrheal agents, including those derived from natural products (e.g., crofelemer, tannic acids, steviodides, etc.) and small molecules identified by using high throughput screening (e.g., CFTRinh-172, GlyH-101, PPQ-102, BPO-27, iOWH032 etc.) [30,39,40]. 

## 3. Strategies to Target CFTR-NHERF2-LPA_2_ Complex for Possible Therapeutic Interventions of CF and Secretory Diarrhea

### 3.1. Strategies to Target CFTR-NHERF2-LPA_2_ Complex for Possible Therapeutic Interventions of CF

Traditional CF therapies target the downstream disease consequences/symptoms, including improving the mucociliary clearance (e.g., restoration of ASL, mucus alteration), anti-inflammatory, anti-infective, improving nutrition, and lung transplantation [41]. Recently, the development of CFTR modulators to restore CFTR function has fundamentally changed CF disease management. Two types of these modulators are potentiators and correctors. CFTR potentiators are molecules that increase the channel gating of CFTR, while CFTR correctors are molecules that rescue the folding and/or trafficking of CFTR to increase the cell surface density. Kalydeco^®^ (ivacaftor, a CFTR potentiator) was approved by U.S. Food and Drug Administration to treat CF patients with G551D and other nine class III and IV mutations (G178R, S549N, S549R, G551S, G1244E, S1251N, S1255P, G1349D and R117H) [42]. Orkambi^®^ (a combination of ivacaftor and lumacaftor; a CFTR corrector) was approved to treat CF patients age 6 years and older with two copies of Phe508del [43].

Based on the findings that (i) LPA levels are elevated in BAL fluid of subjects with CF [36], (ii) CFTR-NHERF2-LPA_2_ complex at the apical plasma membrane of airway epithelial cells functionally couples LPA_2_-mediated signaling to CFTR channel function, and (iii) airway epithelial cells play a central role in regulating mucociliary clearance and modulating the innate and adaptive immune responses after infection [44], we explored the possibility of targeting CFTR-NHERF2-LPA_2_ complex to tackle two major pathologies associated with CF: loss or dysfunction of CFTR channel function, and excessive inflammation.

#### 3.1.1. Disruption of NHERF2-LPA_2_ Interaction to Potentiate CFTR Channel Function

**Figure 3:**
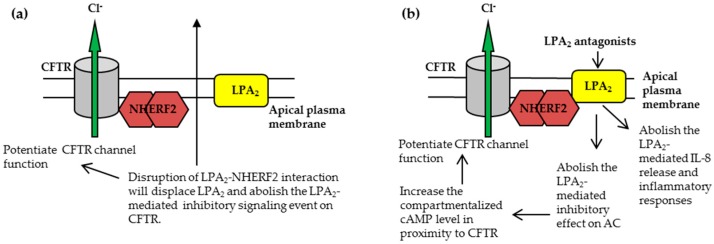
The CFTR-NHERF2-LPA_2_ complex could be targeted to attenuate the LPA_2_-mediated inhibitory signaling on CFTR channel function and/or suppress the LPA_2_-mediated IL-8 release from airway epithelial cells. These two effects are beneficial to CF therapy. (**a**) The approach to disruption of NHERF2-LPA_2_ interaction; (**b**) The approach to antagonism of LPA_2_.

Because disruption of NHERF2-LPA_2_ interaction using an LPA_2_-specific peptide reversed the LPA_2_-mediated inhibition of CFTR channel function [16], one straightforward approach was to develop small-molecule mimics of this LPA_2_ peptide, with more favorable drug-like properties such as improved potency and specificity, bioavailability, and being metabolically stable. As a proof-of-concept study, we developed an amplified luminescent proximity homogeneous assay to screen for compounds that specifically disrupt the LPA_2_–NHERF2 interaction [45]. From a library of 80 available inhibitors (disruptors) of PDZ domain-mediated protein–protein interactions, one hit compound (named CO-068) was found to have the highest potency (IC_50_ = 63 μM) and specificity [45]. Used at 50 μM concentration, CO-068 was found to (i) specifically disrupt the LPA_2_–NHERF2 interaction in cells, without affecting CFTR-NHERF2 or NHERF2-PLC-β3 interactions; (ii) augment the basal CFTR-mediated I_sc_ in polarized Calu-3 cells; (iii) augment the FSK-induced and CFTR-mediated I_sc_ in polarized Calu-3 cells; (iv) increase both the basal and FSK-stimulated CFTR-dependent submucosal glands fluid secretion in an ex vivo pig model, and (v) elevate the compartmentalized cAMP levels in cells [45]. The study was the first to demonstrate that specific disruption of NHERF2-LPA_2_ interaction potentiates the WT-CFTR channel function under the basal and stimulated conditions (Figure 3a).

To explore the possibility of using this approach for CF therapy, we tested the effect of CO-068 on the channel function of the rescued Phe508del-CFTR in two assays. We found that CO-068 potentiated the I_sc_ of the rescued Phe508del-CFTR in polarized CFBEo^−^-Phe508del-CFTR cells and induced the swelling of intestinal enterospheres developed from WT mice and from *Phe508del^−/−^* mice [29]. The data suggests that, when in combination with a CFTR corrector (e.g., lumacaftor), specific disruption of NHERF2-LPA_2_ interaction can potentiate the channel function of Phe508del-CFTR.

Regarding this disruptor approach (Figure 3a), it should be noted that (i) CO-068 has a IC_50_ value of 63 μM, which could account for the moderate potentiating effect on CFTR channel function we observed, and also highlight the need for chemical optimization to discover more potent and specific lead compounds; (ii) because this approach abolishes an LPA_2_-mediated inhibitory signaling on CFTR, in addition to WT- and Ph508del-CFTR, it could augment the channel activities of other CFTR mutants that complex with LPA_2_, therefore representing a mutation non-specific approach; and (iii) more work is needed to investigate the relevance of this approach in CF therapy, e.g., testing its efficacy in primary human CF airway epithelial cells and tissues, toxicological studies, etc.

Another disruptor approach has also been explored to increase the channel activity of Phe508del-CFTR. This approach was designed to disrupt the interaction between CFTR and the CFTR-associated ligand (CAL). CAL is a PDZ domain-containing protein and a negative regulator of Phe508del-CFTR surface abundance [46]. Cushing and colleagues [47] developed a decametric peptide iCAL36 that selectively binds the PDZ domain of CAL. The authors showed that iCAL36 enhanced the functional stability of Phe508del-CFTR and had complementary rescue effect with CFTR corrector corr-4a [47]. This group and their collaborators then used computational approach to study the binding of iCAL36 and derivatives to CAL to develop more specific and efficient inhibitors [48,49]. More recently, they developed a cell-permeable peptidyl inhibitor and demonstrated that this inhibitor disrupted the CFTR/CAL-PDZ interaction and increased the channel activity of Phe508del-CFTR in combination with CFTR correctors [50]. 

Taken together, these disruptor studies provide proof-of-principle data for targeting PDZ domain-mediated protein-protein interactions within the CFTR-containing macromolecular complexes for the possible therapeutic intervention of CF.

#### 3.1.2. Targeting CFTR-NHERF2-LPA_2_ Complex to Suppress the Release of Interleukin 8 (IL-8)

Given the critical role of IL-8 and the IL-8 receptor signaling pathway in the pathogenesis of CF lung inflammation [51], we performed a proof-of-concept study to test whether CFTR-NHERF2-LPA_2_ complex regulates the IL-8 secretion from airway epithelial cells (CFBEo^−^-Phe508del-CFTR cells), thereby playing a role in the pathological cascade of excessive inflammation which fails to eradicate pulmonary pathogens in CF.

We found that (i) LPA_2_ expression level was elevated in CFBEo^−^-Phe508del-CFTR cells compared with that in CFBEo^−^-WT-CFTR cells; (ii) CFBEo^−^-Phe508del-CFTR cells secreted IL-8 under basal (un-stimulated) conditions and responded to IL-1β stimulation to secret large amount of IL-8; (iii) Rescue of Phe508del-CFTR by using lumacaftor slightly decreased the basal level of secreted IL-8 and significantly decreased the IL-1β-stimulated IL-8 secretion (11%); (iv) Low temperature (28 °C) rescue of Phe508del-CFTR dramatically decreased IL-8 secretion from CFBEo^−^-Phe508del-CFTR cells; and (v) Antagonism of LPA_2_ using a specific inhibitor decreased the basal level (44%) and IL-1β-stimulated IL-8 (~39%) secretion [52].

The data suggests that in addition to its role in regulating airway fluid homeostasis, CFTR-NHERF2-LPA_2_ complex may also play a role in modulating IL-8 secretion from airway epithelial cells (Figure 3b). Rescue of Phe508del-CFTR and antagonism of LPA_2_ attenuate the IL-8 release from CFBEo^−^-Phe508del-CFTR cells, which could suppress the initiation of inflammatory response in CF and therefore inhibit the excessive infiltration of neutrophils and the subsequent inflammation. Of course, this hypothesis needs to be tested in other CF cell lines, especially in primary human CF airway epithelial cells, and thereafter in animal studies. In addition, studies are needed to test whether rescue of Phe508del-CFTR and antagonism of LPA_2_ affect the release of other chemokines/cytokines from airway epithelial cells.

Theoretically, antagonism of LPA_2_ will suppress the LPA_2_-mediated G_i_ signaling on AC and increase the compartmentalized cAMP level in proximity to CFTR, and therefore potentiate CFTR channel function. Currently, we are testing if these antagonists potentiate CFTR channel activity in a variety of airway epithelial cell lines.

### 3.2. Targeting CFTR–NHERF2–LPA_2_ Complexes in the Gut Epithelia for Possible Therapeutic Interventions of Secretory Diarrhea

In addition to directly blocking CFTR channel, CFTR-NHERF2-LPA_2_ complex could also be targeted to tackle the abnormal fluid homeostasis in secretory diarrhea. One potential approach is to use LPA, because LPA not only inhibits the CFTR-dependent intestinal fluid secretion through LPA_2_-mediated G_i_ pathway [16], but stimulates the intestinal Na^+^ and fluid absorption by activating NHE3 through an LPA_5_-mediated signaling cascade [53]. These two effects are beneficial to secretory diarrhea therapy. In this regard, LPA-rich food (e.g., hen egg yolk and white, and LPA-enriched soy lipid extract) could be an inexpensive alternative to mitigate secretory diarrhea.

**Figure 4:**
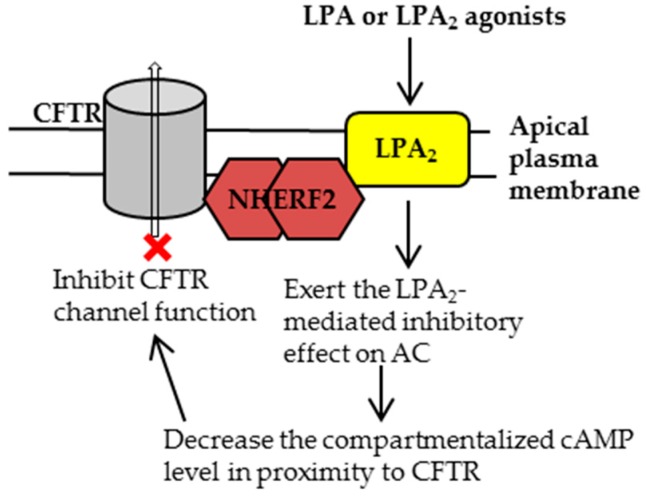
In the gut, the CFTR-NHERF2-LPA_2_ complex could be targeted by using LPA or LPA_2_ agonists to inhibit CFTR channel activity. This inhibition would be beneficial for the therapeutic intervention of secretory diarrhea.

Similarly, it can be envisioned that specific and potent LPA_2_ agonists would inhibit the CFTR channel function and have the potential to inhibit intestinal fluid secretion (Figure 4). As a proof-of-concept study, we tested the efficacy of a specific LPA_2_ agonist, GRI977143 [54], on the CFTR Cl^−^ channel function. We found that (i) GRI977143 significantly inhibited the FSK-induced and CFTR-mediated I_sc_ in polarized human gut epithelial cells (HT29-CL19A cells); (ii) GRI977143 significantly inhibited the CTX-induced and CFTR-dependent intestinal fluid secretion in a closed-loop fluid secretion mouse model; and (iii) GRI977143 inhibited both the basal and FSK-induced cAMP levels at the plasma membrane of HT29-CL19A cells [55]. Currently, we are testing whether GRI977143 affects the IL-8 and other chemokines/cytokines release from intestinal epithelial cells and whether GRI977143 affects the integrity of tight junction in these cell lines.

## 4. Conclusions

CF and secretory diarrhea are two major human diseases associated with dysregulated CFTR channel activities. Since the discovery of the *CFTR* gene in 1989, significant progress has been made in understanding the CF pathogenesis and in development of effective CF therapies. The approval of Kalydeco^®^ and Orkambi^®^ has proven that CFTR is a validated drug target.

Because the channel function of CFTR is regulated by dynamic protein–protein interactions in a complex network, identification of these CFTR-containing macromolecular complexes can further our understanding of CFTR biology in health and in disease, and open up potential avenues for drug development to treat CFTR-associated diseases. Accumulating evidence suggests that CFTR complexes with NHERF2 and LPA_2_ at the apical plasma membrane of airway and gut epithelial cells, which couples the LPA_2_-mediated signaling to CFTR channel function. Several approaches could be explored to target this complex to fine tune CFTR channel activity, including using specific disruptors of LPA_2_–NHERF2 interaction and specific LPA_2_ modulators. With the technological advancements in drug discovery, we believe that more potent and specific small molecules will be identified and tested, which will provide us more tools to gain insights into the pathophysiology of CFTR and to combat CFTR-associated diseases.

## Abbreviations

ABC	ATP-binding cassette
AC	Adenylyl cyclase
β_2_-AR	β_2_-Adrenergic receptor
ASL	Airway surface liquid
BAL	Bronchoalveolar lavage
CAL	CFTR-associated ligand
CF	Cystic fibrosis
CFBEo^−^-	CF bronchial epithelial cells
CFTR	CF transmembrane conductance regulator
CTX	Cholera toxin
ERM	Ezrin/radixin/moesin
FSK	Forskolin
IL-8	Interleukin 8
I_sc_	Short-circuit currents
LPA	Lysophosphatidic acids
LPA_2_	LPA receptor 2
MRP4	Multidrug resistance protein 4
MSD	Membrane-spanning domain
NBD	Nucleotide binding domain
NHE	Na^+^/H^+^ exchanger
NHERF2	Na^+^/H^+^ exchanger regulatory factor 2
PDZ	Postsynaptic density-95, discs large, zona occludens-1
PLC-β3	Phospholipase C-β3
R domain	Regulatory domain
WT	Wild type
